# National Seroprevalence and Risk Factors of Bluetongue Virus in Domestic Ruminants of Peru

**DOI:** 10.1155/tbed/2690231

**Published:** 2025-01-10

**Authors:** Dennis A. Navarro-Mamani, Jessica Jurado, Ana Vargas-Calla, Kevin Ponce, Tyler Sherman, Yari Zarate, César A. Murga-Moreno, Ibelice Perez, Ruben Villacaqui, Miguel Ara, Pedro Ortiz, Hermelinda Rivera, Christie E. Mayo

**Affiliations:** ^1^Laboratory of Virology, Faculty of Veterinary Medicine, National University of San Marcos, Lima, Peru; ^2^Laboratory of Parasitology, Faculty of Veterinary Medicine, National University of San Marcos, Lima, Peru; ^3^Department of Microbiology, Immunology, and Pathology, Colorado State University, Fort Collins, Colorado, USA; ^4^Faculty of Agronomy and Animal Science, National University of San Antonio Abad of Cusco, Cusco, Peru; ^5^Tropical Medicine Research Center, Faculty of Veterinary Sciences, National University of Cajamarca, Cajamarca, Peru; ^6^Servicio Nacional de Sanidad Agraria (SENASA), Lima, Peru; ^7^Laboratory of Biochemistry, Nutrition, and Animal Feeding, Faculty of Veterinary Medicine, National University of San Marcos, Lima, Peru

**Keywords:** antibodies, bluetongue, cELISA, risk factors, true seroprevalence

## Abstract

Bluetongue (BT) is a viral infection caused by the bluetongue virus (BTV) that affects domestic and wild ruminants worldwide. It is primarily transmitted by *Culicoides* spp. midges, and its infection is highly prevalent across temperate and tropical regions. However, significant changes in the global distribution of BTV have been observed in recent years. We aimed to evaluate the national BTV seroprevalence and risk factors among domestic ruminants (cattle, sheep, and goat species) in Peru. Serum samples were collected from 3452 cattle of 453 districts, 2786 sheep of 408 districts, and 1568 goats of 271 districts using a cross-sectional study in two stages (at the district and animal level) from 2017 to 2019 and analyzed by competitive enzyme-linked immunosorbent assay (cELISA). The national BTV true seroprevalences at animal level for cattle, sheep, and goats were 20.34% (95% CI: 17.76–20.82), 7.63% (95% CI: 7.17–9.56), and 8.58% (95% CI: 7.52–10.85), while the true districts-level seroprevalences were 31.53% (95% CI: 24.06–33.77), 24.41% (95% CI: 18.06–27.53), and 13.35% (95% CI: 8.59–17.98), respectively. In addition, we found that altitude and maximum temperature were identified as important factors influencing the seroprevalence of BTV in cattle, sheep, and goats. Higher altitudes above 3000 m above sea level (masl) played a protective role, reducing the BTV seroprevalence. In conclusion, antibodies against BTV were detected in Peruvian domestic ruminants without clinical signs. The seroprevalence was low in the South (<10.0%), varied in the Center and North, and high (>30%) in the East (Amazon rainforest). This study lays the groundwork for identifying BTV serotypes and *Culicoides* spp. in different regions, including altitudes above 3000 masl, to enhance BTV surveillance in Peru.

## 1. Introduction

Globally distributed arboviruses are a severe threat to animal and public health, as it has been evidenced with the emergence and reemergence of epidemic arboviral diseases [1]. Bluetongue (BT) disease occurs in sporadic outbreaks with economic importance that affect both wild and domestic ruminants worldwide. Bluetongue virus (BTV), the causative agent of BT, is a vector-borne virus transmitted by *Culicoides* species, small biting insects with ranges distributed between latitudes –35°S and 40°–50°N. BT is endemic where competent *Culicoides* species and favorable climatic conditions are presented in the same geographical space [2, 3]. In addition, the potential for vectors to migrate into areas outside of their natural range due to climatic and ecological modifications can result in further epidemics of BT [4, 5]. Consequently, previous epidemics have caused global economic damage with impacts upwards of $3 billion USD due to decreased animal production, international trade restrictions of live animals and their by-products, and increasing costs of surveillance and control measures [6, 7].

BTV is the prototype species of the *Orbivirus* genus, belonging to the *Sedoreoviridae* family. BTV has linear, double-stranded RNA (dsRNA) genomes divided into 10 segments that can undergo segment reassortment, the main ostensible driver of novel serotype emergence in different geographical regions [8]. To date, more than 28 distinct BTV serotypes have been recognized, and their geographic distribution differs from each other [9, 10]. Recently, a BTV-3 epidemic in the Netherlands affected 6000 farms, killing tens of thousands of sheep, and its mortality rates were up to 75%, over 10 times higher than during the BTV-8 outbreak [11]. In the case of South American countries, such as Brazil, Argentina, and Ecuador, some BTV serotypes have been reported, including BTV-1, BTV-2, BTV-3, BTV-4, BTV-9, BTV-12, BTV-13, BTV-18, BTV-19, BTV-22, and BTV-26, with and without clinical signs [12–14]. Although *Culicoides* species are mainly responsible for BTV [2, 15, 16], horizontal and vertical transmission have also been recorded [17–19]. The severity of BT disease varies based on animal species, breed, age, and immune status, as well as the presence of competent *Culicoides* and associated environmental factors [20]. An acute clinical phase of infection is frequently reported in sheep, while cattle and goats are usually either asymptomatic or develop mild symptoms. In cattle, viremia may be prolonged for up to 60 days, making this species a presumptive viral reservoir [21, 22].

South America countries (Brazil, Argentina, Ecuador, Peru, and Chile) do not vaccinate domestic ruminants against BT disease, but have serologically described BTV infection in cattle, sheep, goats, and South American camelids (SAC) [23–25]. In Brazil, 64.8% seroprevalence has recently been reported from Paraná [26] and 56.4% from Minas Gerais in sheep [27]. In Ecuador, 99.1% seroprevalence was recorded in many states from Manabí in cattle [28]. In Peru, BTV seroprevalence has been recorded with a frequency higher than 50% in sheep from Ucayali [29] and Junin et al. [30], and 23.8% in goats from the Peruvian North [31], although there has not been officially reported evidence of clinical BT until now. Since 1998, Europe has faced sporadic incursions of BTV from other areas and its epidemiology has changed dramatically in the last years, resulting in outbreaks in many countries where it has never been seen before [32, 33]. In China, BTV typically affects cattle from intensive farming systems and its presence was also higher in buffalos than dairy cows [34]. Entry of new cattle into herds was noted as another risk factor that was associated with the prevalence of BTV in Brazilian herds [13]. In addition, *Culicoides* species are more abundant between 20 and 25°C which refers to spring and summer seasons, so temperature and precipitation play a key role in BT epidemiology [35].

Currently, Peru has not reported any BT outbreaks, but serological and molecular evidence was recorded in asymptomatic sheep and cattle from the Amazonian rainforest where *Culicoides insignis* was also registered; therefore, BTV is likely actively circulating. Furthermore, according to predictive analytics, *C. insignis* has the potential to invade the Andes, where it may encounter immunologically naive host populations and cause outbreaks in susceptible animals [4, 36, 37]. The aim of this study was to determine the national seroprevalence of BTV antibodies in domestic ruminants (cattle, sheep, and goats) of Peru, while also examining risk factors associated with antibody detection. The results of these examinations will be used to better understand the current and future epidemiology of BTV in Peru and provide impetus for the continuation of active surveillance efforts.

## 2. Methods

### 2.1. Study Area

**Figure 1:**
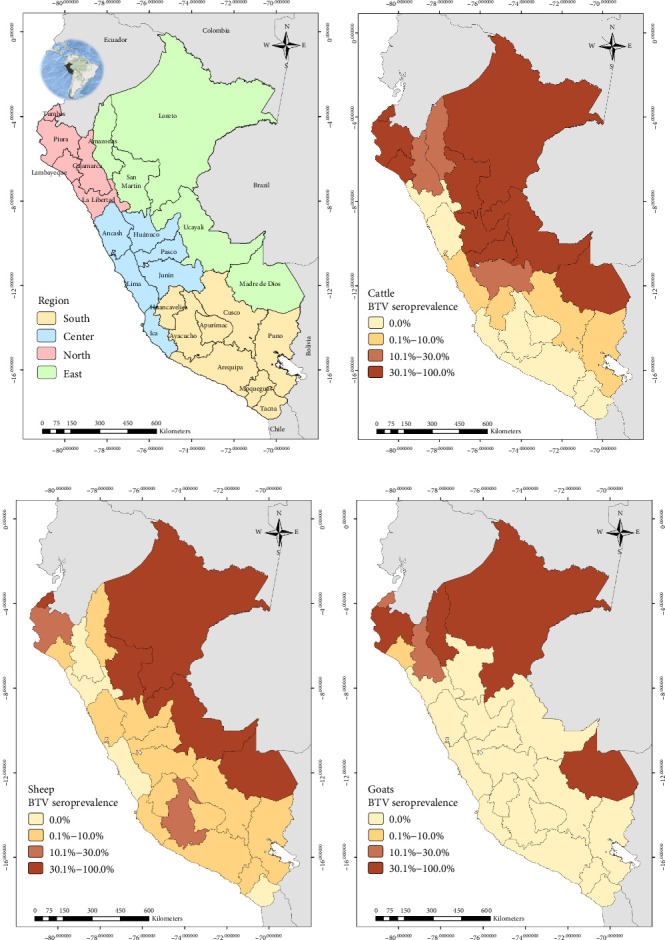
Maps of Peru illustrating the four regions and its 24 departments (A). Additionally, BTV seroprevalence at the animal level is represented using color scales to indicate percentages in cattle (B), sheep (C), and goats (D) across the Peruvian territory. BTV, bluetongue virus.

Peru is in the Central-Western region of South America. It encompasses an area of 1,285,216 km^2^ and is divided into 24 departments and 1874 districts. Also, there are four regions (North, South, Center, and East) based on the geographic classification stipulated by the Servicio Nacional de Sanidad Agraria del Peru (SENASA-Peru) (Figure 1A). According to the Instituto Nacional de Estadística e Informática (INEI), cattle farming is widely established in the South, North, and Center regions (*Supporting Information 1*: Figure S1B). Sheep farming is primarily developed in the South, although some operations exist in the Center and North (*Supporting Information 1*: Figure S1C), while goats are mostly established on the coast of the North and Center regions (*Supporting Information 1*: Figure S1D). Less than 2% of the ruminants are kept in the Peruvian East region. There is no vaccination program for BTV control in Peru.

### 2.2. Sampling Design and Serum Collection

A sampling design was developed and carried out by SENASA-Peru from 2017 to 2019. To estimate the true and apparent seroprevalence of positive districts and animals, a cross-sectional study was developed in two stages. First, a random selection of districts was produced, which represented the primary sampling units. Within these primary units, ruminants >6 months were randomly selected (secondary sampling units) to determine the historical BTV seroprevalence of the species (cattle, sheep, and goats) in each district. A power analysis was conducted using the following parameters: confidence level of 95%, estimated apparent prevalence of 56.1% [30] a standard error of 10%, and an intracluster correlation coefficient of 0.4 [38, 39]. In total, serum samples were collected from 3452 cattle in 453 districts, 2786 sheep in 408 districts, and 1568 goats in 271 districts.

### 2.3. Data Collection

Animal sex (female or male) and age (>6–≤12 months; >12–≤24 months or >24 months) were recorded with each serum sample collection. In addition, meteorological data was obtained from The POWER Project—NASA using the farm's geographical coordinates, such as altitude (≤1000 m above sea level (masl); >1000–≤2000 masl; >2000–≤3000 masl; or >3000 masl—Figure 1B), precipitation (0–≤2 mm/day; >2 mm/day), relative humidity (≤60%; >60%–≤80% or >80%/day), and maximum temperature (≤20°C; >20–≤30°C or >30°C/day) were collected during the study period.

### 2.4. Detection of Antibodies Against BTV

Antibodies specific against the VP7 protein (*Orbivirus* group antigen) of BTV were detected using a commercial competitive enzyme-linked immunosorbent assay (cELISA) kit (ID Screen Bluetongue Competition, ID-Vet, Montpellier, France), according to the manufacturer's instructions. The optical density values were measured in a spectrophotometer (Biotek Service & Supplies S.A.) with a 450-nm filter for a percentage of competition (S/N%) calculation. Serum samples with S/N% values less than 40% were considered positive. Regarding the diagnostic performance of the ID-Vet cELISA kit used in this study, Vandenbussche et al. [40] evaluated the accuracy and reported 87.8% sensitivity (Se) and 98.2% specificity (Sp).

### 2.5. Statistical Analysis

Data from serologic analysis was input into STATA Statistical Software v15 (Stata Corp, College Station, TX, USA) for both descriptive and statistical analyses. The apparent seroprevalence of BTV at the individual animal level was calculated as the proportion of seropositive cattle, sheep, and goats to the total number of examined animals in each species. The district-level seroprevalence was estimated from the ratio of positive districts to the total number of districts tested. Districts containing at least one seropositive cow, sheep, or goat were considered as positive. To obtain the true seroprevalence, both district and individual animal (cattle, sheep, and goats) seroprevalence were adjusted for Se and Sp of the cELISA test. This adjustment was achieved through the application of the formula described by Dohoo, Martin, and Stryhn [41].

Logistic regression analysis was used to evaluate risk factors associated with BTV antibody detection and to estimate odds ratios (OR) with 95% CI. In this sense, serologic status was considered as the main dependent variable, with age, sex, altitude, precipitation, relative humidity, and maximum temperature as independent variables. Initially, bivariate logistic regression was performed to estimate the association of the independent variables with seroprevalence and then a multiple logistic regression model was formulated including all. The model was adjusted to account for the two stages of analyses by using the survey (svy) command in STATA program and setting districts as the primary sampling unit. Separate analyses were conducted for each species of ruminant, and significance for all analyses was determined at a *p*-value of less than 0.05.

## 3. Results

### 3.1. Seroprevalence at the Animal—and District Level

The national BTV seroprevalence among ruminants from Peru was 12.7% (95% CI: 11.0%–14.6%). In general, the seroprevalence at animal level based on the region was lower than 10.0% in the South and higher than 30.0% in the East for the three species. In the case of the North, it was higher than 19% for cattle and goats (Figure 1, Table 1)

Out of the 3,452 cattle tested in this study, 666 were seropositive to BTV with apparent individual animal-level seroprevalence of 19.3% (95% CI: 18.0%–20.7%). At the district level, 131 districts had at least one BTV seropositive cow, giving an apparent district-level seroprevalence of 28.9% (95% CI: 24.8%–33.3%) (Table 1). For sheep, 233 were seropositive to BTV out of 2,786 tested (apparent seroprevalence of 8.4%; 95% CI: 7.4%–9.5%). At the district level, 93 sheep districts had at least one BTV seropositive sheep (apparent district-level seroprevalence of 22.8%; 95% CI: 18.8%–27.2%) (Table 1). Among the tested goats, 144 showed seropositivity to BTV out of a total of 1568, indicating an apparent seroprevalence at the goat level of 9.2% (95% CI: 7.8%–10.7%). Thirty-six districts had at least one seropositive goat (district-level seroprevalence of 13.3%; 95% CI: 9.5%–17.9%).

The apparent animal and district seroprevalence were adjusted to the ELISA test Se (87.8%) and Sp (98.2%). Thus, the true cattle, sheep, and goat-level seroprevalences were 20.34% (95% CI: 17.76–20.82), 7.63% (95% CI: 7.17–9.56), and 8.58% (95% CI: 7.52–10.85), respectively. For both cattle, sheep, and goats, the true district-level seroprevalences were 31.53% (95% CI: 24.06–33.77), 24.41% (95% CI: 18.06–27.53), and 13.35% (95% CI: 8.59–17.98), respectively.

In addition, seroprevalence for each of the 24 departments, which are grouped into regions based on the SENASA, were uploaded in supporting information (*Supporting Information 2*: Table S1).

### 3.2. Risk Factor Analysis

#### 3.2.1. Cattle

**Figure 2:**
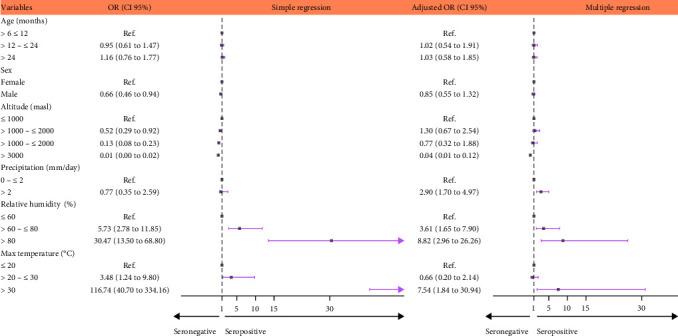
Forest plot of the risk factors among cattle associated with BTV antibody detection in Peru, 2017–2019 by simple and multiple logistic regression. BTV, bluetongue virus.

Bivariate analysis indicated that increased precipitation, relative humidity, and maximum temperature were all associated with BTV antibody detection. On the other hand, the male sex and increasing altitudes were associated with decreases in antibody detection. According to the multiple logistic regression, cattles at altitudes greater than 3,000 masl were 0.04 (*p* < 0.001) times less likely to have BTV antibodies than animals at altitudes lower than 1,000 masl. In addition, we found that precipitation greater than 2 mm/day (*OR* = 2.90, *p* < 0.001), relative humidity from 60% to 80%, and/or over 80% (*OR* = 3.61, *p*=0.001 and *OR* = 8.82, *p* < 0.001, respectively), and maximum temperatures greater than 30°C (*OR* = 7.54, *p*=0.005) were all significantly associated with BTV antibody seroprevalence (Figure 2, *Supporting Information:* Table S2).

#### 3.2.2. Sheep

**Figure 3:**
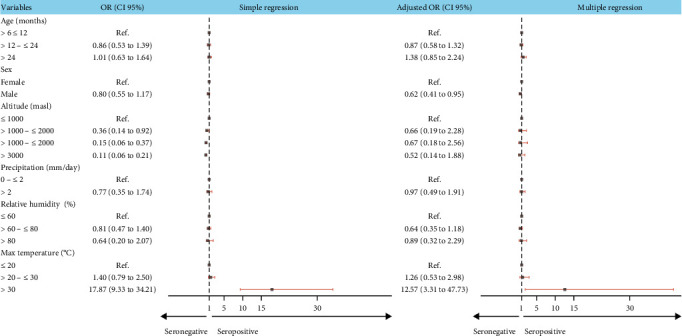
Forest plot of the risk factors among sheep associated with BTV antibody detection in Peru, 2017–2019 by simple and multiple logistic regression. BTV, bluetongue virus.

The results of simple logistic regression indicated that altitude was negatively associated, while the maximum temperature was positively associated with BTV seroprevalence. The multiple logistic regression confirmed that temperatures greater than 30°C are a risk factor for BTV (*OR* = 12.57, *p* < 0.001). Also, we found that male sheep were 0.62 (*p*=0.029) times less likely to be BTV antibodies positive than females (Figure 3, *Supporting Information 2*: Table S3).

#### 3.2.3. Goats

**Figure 4:**
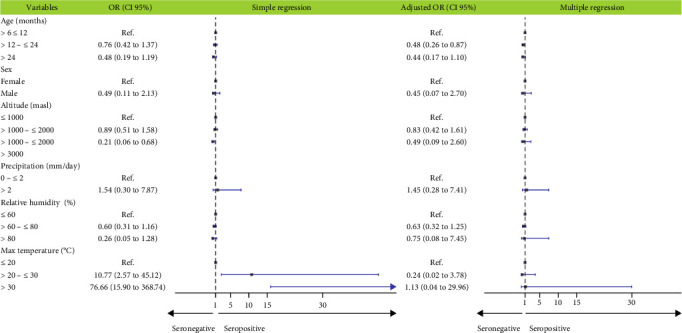
Forest plot of the risk factors among goats associated with BTV antibody detection in Peru, 2017–2019 by simple and multiple logistic regression. BTV, bluetongue virus.

Bivariate analysis indicated that maximum temperature was associated with increased BTV antibody detection, while the increased altitude was associated with decreased detection. The results of multiple logistic regression showed that only age was significantly associated with BTV seroprevalence: goats between 12 and 24 months were 0.48 (*p*=0.016) times less likely to be BTV seropositive than goats younger than 12 months (Figure 4, *Supporting Information 2*: Table S4).

## 4. Discussion

The work described here represents the first official study conducted to determine the seroprevalence of BTV in cattle, sheep, and goats in Peru. BTV seroprevalence among all ruminants was found to be 12.7% (95% CI: 11.0%−14.6%). Additionally, the study revealed varying results across different regions and departments, suggesting a possible correlation between the predominant type of animal husbandry practices and the corresponding ecosystems in those areas. For instance, transhumant-type goat that is an extensive farming system where herds are moved to uplands areas in order to exploit the rangelands is a predominant practice in the North where the main ecosystem is characterized by dry forests. In contrast, the Northern Andean region is characterized by warm to cold climates and provides favorable conditions for both livestock development and *Culicoides* presence [42]. The largest sheep and cattle populations are raised in the central and Southern Andean mountains (located at altitudes between 2500 and 4500 masl), and the environmental conditions in this region are likely less favorable for the *Culicoide*s BTV vectors. This might explain the lower BTV seroprevalence observed in these areas compared to the results obtained in the Amazon rainforest (East region) and the Peruvian North.

The detection of antibodies in animals for this study indicates true exposure to BTV given that Peruvian ruminants are not vaccinated against BTV (this includes restrictions against the importation of vaccines on individual farms). Seropositivity against BTV in ruminants from the North and Peruvian Amazon rainforest (East) ranged between 19.1% and 100%, while the antibodies detected in ruminants in Central and southern regions were lower. The central and southern regions harbor the largest livestock populations, characterized by diverse ecological environments and temperatures [43]; however, the presence of antibodies against BTV in animals from these regions may be attributed to the incursion of midge vectors depending on factors such as the season of the year, climate, precipitation, breeding altitude, and management practices [44]. Similar results were reported in populations of yaks from the Tibetan plain in China, where BTV antibody prevalence ranges from 2% to 6% at altitudes over 3,000 masl due to presumptive incursions of infected midges during the short summer season [45, 46].

The seroprevalence of BTV antibodies detected in ruminants from Peru was lower than what has been reported in other Latin American countries where BTV is endemic. For instance, cattle showed seroprevalences from 98% to 100% in Ecuador [14, 28], whereas cattle from Minas Gerais, Brazil, were reported with 83.3% seroprevalence [47]. Cattle from Venezuela were reported with 74.8% of seroprevalence in one study [48]. The variance in these results between Peru and other countries, like Ecuador, may be reflective of the sampling strategy used (i.e., results reported here include sampling from the entire country of Peru) or that the Andean highlands could act as a natural barrier, restricting the migration of *Culicoides* to the coastal regions of Peru. On the other hand, factors such as altitude, temperature, and precipitation influence the presence of *Culicoides* species in the Peruvian Amazon rainforest (East region), where seroprevalence for BTV is higher [44].

Results of the multiple regression analysis regarding altitude revealed that altitudes higher than 3000 masl were associated with decreased antibody detection. Indeed, altitude is undoubtedly a protective factor against BTV seroprevalence, possibly limiting *Culicoides* incursions during favorable conditions though, as indicated by some authors [45, 46]. In recent decades, global warming may have facilitated the migration of *Culicoides* species from warmer ecosystems to higher altitudes, where susceptible animal populations can be found. In contrast to the lower altitudes, sheep are raised in the central and southern regions of the country, specifically in the Andean region (>3000 masl). The Andes Highlands provides favorable conditions for sheep farming, including suitable grazing areas, moderate to low temperatures, and access to fresh water sources in a semi-extensive system. We obtained that higher altitudes than 1,000 masl were negatively associated with BTV, and seroprevalence was also low from 3.7% to 7.7% in the Center and South, respectively. *Culicoides* midges' incursions could be associated with management activities in sheep, including shearing due to the seasonal nature of this practice. In the central highlands, shearing typically takes place in March, which marks the beginning of autumn, while in the southern regions, this management practice occurs in November or December during the summer season. It is important to note that these periods may coincide with different climatic conditions. In the central highlands, March is generally drier, while in the south, it can be a rainy season. The presence of rainfall can create suitable breeding habitats for *Culicoides* vectors, potentially increasing the risk of BTV transmission during those times [49]. However, further research is needed to investigate the potential impact of sheep management practices on BTV infection and transmission risks.

Lastly, our study revealed that temperatures above 30°C were identified as a risk factor for BTV seroprevalence. It is important to note that high seroprevalence of BTV in sheep without clinical signs was found in the Amazon rainforest (East region) where BTV is considered endemic. It is evident that temperatures ranging from 20 to 25°C during spring and summer, along with factors such as precipitation, wind speed, and rainfall, influenced the abundance of *Culicoides*, which are associated with BTV infection [16, 50]. The Amazonian rainforest, located within the Neotropics and the Amazon basin, comprises tropical and subtropical moist broadleaf forests [51], providing an endemic environment for BTV due to the presence of its *Culicoides* vector [1]. The favorable temperature conditions in this region facilitate rapid BTV replication, shorten the intervals between *Culicoides* feedings, and increase the risk of transmission. Additionally, higher temperatures accelerate the development of *Culicoides* eggs and larvae, while potentially impacting the lifespan of female *Culicoides* [16, 52, 53].

Compelling studies on global warming and climate change indicate that their effects will have a significant impact on public health and animal health in different regions of South America [36, 37, 54]. Peru is one of the countries most vulnerable to climate change, and certain events such as El Niño–Southern Oscillation (ENSO) phenomenon could become more intense, creating favorable conditions for the distribution and survival of *Culicoides* vectors of BTV in the Andean region, especially in the North. While clinical cases of BTV disease have not been reported to date, it is necessary to identify the BTV infection prevalence and circulating serotypes, as well as the species of the *Culicoides* genus, including abundance and ecological niche of *C*. *insignis* that is present in different ecosystems of the country. These data are essential for active serological and molecular surveillance by the Peruvian health authorities.

## 5. Conclusions

This study provides basic epidemiological information on BTV in domestic ruminants in Peru. BTV exposition is widespread nationwide in cattle, sheep, and goats. BTV seroprevalence was low in the South, varied in the Center and North, and high in the East (Amazon rainforest) regions. Risk factors associated with BTV antibody detection varied among the evaluated ruminant species and their respective regions of origin. The study serves as a foundation for further identifying circulating BTV serotypes nationwide and identifying *Culicoides* species in different regions, including altitudes above 3000 masl, as an active surveillance of BTV in Peru's major livestock-populated areas. Although there is currently no evidence of clinical disease in ruminants in the country, this active surveillance provides a framework to better document and understand current epidemiology and epidemics that could potentially occur in the near future due to the ramifications of climate change.

## Figures and Tables

**Table 1:** Apparent seroprevalence of districts and animal (cattle, sheep, and goats) positive for bluetongue virus in the four regions of Peru, 2017–2019.

Regions	Cattle				Sheep				Goat			
	*n*	Positive	%	95% CI	*n*	Positive	%	95% CI	*n*	Positive	%	95% CI
*Animal level*												
South	1862	9	0.4	0.2–0.9	1408	109	7.7	6.4–9.3	544	0	0.0	0.0–0.8
Center	481	100	20.8	17.3–24.7	874	32	3.6	2.5–5.1	330	0	0.0	0.0–1.1
North	704	236	33.5	30.0–37.1	417	35	8.4	5.9–11.5	666	127	19.1	16.2–22.3
East	405	321	79.3	74.9–83.1	87	57	65.5	54.6–75.4	28	17	60.7	40.6–78.5
Total	3452	666	19.3	18.0–20.7	2786	233	8.4	7.4–9.5	1568	144	9.2	7.8–10.7
*District level*												
South	171	6	3.5	1.3–7.5	192	43	22.4	16.7–28.9	104	0	0.0	0.0–3.5
Center	92	26	28.3	19.4–38.6	123	18	14.6	8.9–22.1	65	0	0.0	0.0–0.6
North	147	62	42.2	34.1–50.6	68	13	19.1	10.6–30.5	94	29	30.9	21.7–41.2
East	43	37	86.1	72.1–94.7	25	19	76.0	54.9–90.7	8	7	87.5	47.3–99.7
Total	453	131	28.9	24.8–33.3	408	93	22.8	18.8–27.2	271	36	13.3	9.5–17.9

*Note:* %, seroprevalence

Abbreviations: CI, confidence interval; n, number of animals.

## Data Availability

The data that support the findings of this study, including those in the supporting information, are available from the corresponding author upon reasonable request.

## Supporting Information

Supporting Information 1 Figure S1: Maps of Peru illustrating the four regions and its 24 departments (A). Additionally, district-level ruminant populations are depicted using color scales to represent the number of cattle (B), sheep (C), and goats (D) across the Peruvian.

Supporting Information 2 Table S1: Seroprevalence of bluetongue virus at animal-level in domestic ruminants (cattle, sheep and goats) by department of Peru, 2017–2019. Table S2: Seroprevalence of bluetongue virus at animal level in domestic ruminants (cattle, sheep, and goats) by department of Peru, 2017–2019. Table S3: Potential risk factors among sheep for BTV antibody detection in Peru, 2017–2019 by simple and multiple logistic regression. Table S4: Potential risk factors among goats for BTV antibody detection in Peru, 2017–2019 by simple and multiple logistic regression.
