# Supplementary material for: National Seroprevalence and Risk Factors of Bluetongue Virus in Domestic Ruminants of Peru
Source: Transbound Emerg Dis. 2025 Jan 10;2025:2690231. doi: 10.1155/tbed/2690231 (PMC12016983; doi:10.1155/tbed/2690231)
Supplement: Supporting Information 1 — Figure S1: Maps of Peru illustrating the four regions and its 24 departments (A). Additionally, district-level ruminant populations are depicted using color scales to represent the number of cattle (B), sheep (C), and goats (D) across the Peruvian. [file 2690231.f1.docx]

**Supplementary 1 Figure 1:**

The maps show the distribution of cattle, sheep, and goat population across Peruvian territory. Cattle are primarily raised in the Andean and coastal regions. Sheep are mainly found in the northern Andean region (brown). Goats are predominantly raised in the coastal regions of central and northern Peru.


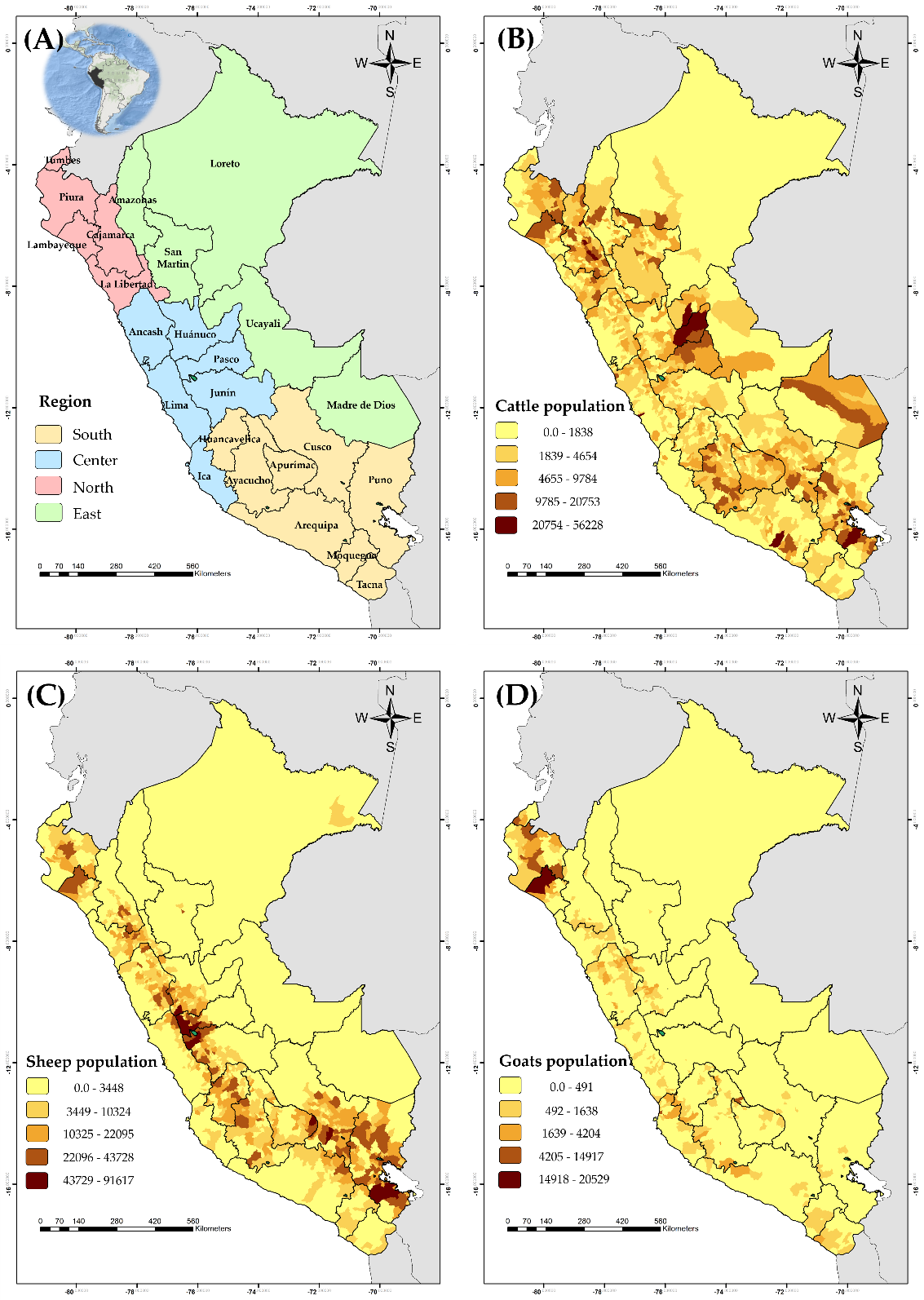


**Figure 1.** Maps of Peru illustrating the four regions and its 24 departments (A). Additionally, district-level ruminant populations are depicted using color scales to represent the number of cattle (B), sheep (C), and goats (D) across the Peruvian territory
