# Supplementary material for: National Seroprevalence and Risk Factors of Bluetongue Virus in Domestic Ruminants of Peru
Source: Transbound Emerg Dis. 2025 Jan 10;2025:2690231. doi: 10.1155/tbed/2690231 (PMC12016983; doi:10.1155/tbed/2690231)
Supplement: Supporting Information 2 — Table S1: Seroprevalence of bluetongue virus at animal-level in domestic ruminants (cattle, sheep and goats) by department of Peru, 2017–2019. Table S2: Seroprevalence of bluetongue virus at animal level in domestic ruminants (cattle, sheep, and goats) by department of Peru, 2017–2019. Table S3: Potential risk factors among sheep for BTV antibody detection in Peru, 2017–2019 by simple and multiple logistic regression. Table S4: Potential risk factors among goats for BTV antibody detection in Peru, 2017–2019 by simple and multiple logistic regression. [file 2690231.f2.docx]

**Supplementary 2 Table 1:**

The following table shows the seroprevalence of the bluetongue virus across different departments in Peru. Peru has 24 departments (political divisions), which are grouped into four based on the Servicio Nacional de Sanidad Agraria (SENASA)

**Table 1.** Seroprevalence of bluetongue virus at animal-level in domestic ruminants (cattle, sheep and goats) by department of Peru, 2017-2019

| Region/  Department | | Bovine | | | |  | Sheep | | | |  | Goat | | | |
| --- | --- | --- | --- | --- | --- | --- | --- | --- | --- | --- | --- | --- | --- | --- | --- |
|  |  | n | seropositive | % | CI 95% |  | n | seropositive | % | CI 95% |  | n | seropositive | % | CI 95% |
| North | |  |  |  |  |  |  |  |  |  |  |  |  |  |  |
|  | Tumbes | 34 | 29 | 85.3 | 48.8 - 97.2 |  | 24 | 9 | 37.5 | 6.8 - 83.1 |  | 98 | 21 | 21.4 | 8.8 - 43.4 |
|  | Piura | 137 | 101 | 73.7 | 63.3 - 82.0 |  | 88 | 24 | 27.3 | 12.9 - 48.6 |  | 266 | 89 | 33.5 | 14.9 - 59.0 |
|  | Lambayeque | 29 | 12 | 41.4 | 12.3 - 78.1 |  | 36 | 2 | 5.6 | 1.0 - 25.2 |  | 80 | 3 | 3.8 | 0.5 - 23.6 |
|  | Cajamarca | 366 | 94 | 25.7 | 17.3 - 36.3 |  | 135 | 0 | 0 | - |  | 134 | 14 | 10.4 | 5.3 - 19.6 |
|  | La Libertad | 138 | 0 | 0 | - |  | 134 | 0 | 0 | - |  | 88 | 0 | 0 | - |
| Center | |  |  |  |  |  |  |  |  |  |  |  |  |  |  |
|  | Ancash | - | - | - | - |  | 212 | 2 | 0.9 | 0.1 - 6.9 |  | - | - | - | - |
|  | Huánuco | 130 | 57 | 43.9 | 25.3 - 64.2 |  | 208 | 16 | 7.7 | 1.9 - 26.9 |  | 84 | 0 | 0 | - |
|  | Lima-Callao | 121 | 3 | 2.5 | 0.8 - 7.2 |  | 130 | 0 | 0 | - |  | 66 | 0 | 0 | - |
|  | Pasco | 46 | 25 | 54.4 | 23.5 - 82.2 |  | 112 | 6 | 5.4 | 3.0 - 9.3 |  | 16 | 0 | 0 | - |
|  | Junín | 134 | 15 | 11.2 | 4.4 - 25.6 |  | 184 | 6 | 3.3 | 1.4 - 7.6 |  | 44 | 0 | 0 | - |
|  | Ica | 50 | 0 | 0 | - |  | 28 | 2 | 7.1 | 0.9 - 40.6 |  | 120 | 0 | 0 | - |
| South | |  |  |  |  |  |  |  |  |  |  |  |  |  |  |
|  | Huancavelica | 68 | 3 | 4.4 | 0.8 - 20.7 |  | 176 | 15 | 8.5 | 4.3 - 16.3 |  | 106 | 0 | 0 | - |
|  | Ayacucho | 371 | 0 | 0 | - |  | 176 | 41 | 23.3 | 10.5 - 43.9 |  | 164 | 0 | 0 | - |
|  | Apurímac | 294 | 0 | 0 | - |  | 144 | 5 | 3.5 | 1.2 - 9.4 |  | 50 | 0 | 0 | - |
|  | Arequipa | 120 | 0 | 0 | - |  | 26 | 1 | 3.8 | 0.2 - 48.0 |  | 76 | 0 | 0 | - |
|  | Cusco | 228 | 4 | 1.8 | 0.5 – 4.4 |  | 360 | 23 | 6.4 | 2.9 - 13.6 |  | 76 | 0 | 0 | - |
|  | Puno | 696 | 2 | 0.3 | 0.1 - 1.1 |  | 482 | 22 | 4.6 | 2.6 - 8.0 |  | 16 | 0 | 0 | - |
|  | Moquegua | 34 | 0 | 0 | - |  | 24 | 2 | 8.3 | 0.2 - 77.8 |  | 20 | 0 | 0 | - |
|  | Tacna | 51 | 0 | 0 | - |  | 20 | 0 | 0 | - |  | 36 | 0 | 0 | - |
| East | |  |  |  |  |  |  |  |  |  |  |  |  |  |  |
|  | Amazonas | 80 | 9 | 11.3 | 3.3 - 32.3 |  | 24 | 2 | 8.3 | 0.5 - 61.7 |  | 16 | 5 | 31.3 | 8.3 - 69.5 |
|  | Loreto | 68 | 60 | 88.2 | 73.9 - 95.2 |  | 8 | 6 | 75 | - |  | 4 | 4 | 100 | - |
|  | Madre de Dios | 70 | 70 | 100 | - |  | 18 | 18 | 100 | - |  | 8 | 8 | 100 | - |
|  | San Martin | 144 | 139 | 96.5 | 77.6 - 99.6 |  | 12 | 7 | 58.3 | 12.7 - 93.1 |  | - | - | - | - |
|  | Ucayali | 43 | 43 | 100 | - |  | 25 | 24 | 96 | 69.6 - 99.6 |  | - | - | - | - |
| Total | | 3452 | 666 | 19.3 | 18.0 - 20.7 |  | 2786 | 233 | 8.4 | 7.4 - 9.5 |  | 1568 | 144 | 9.2 | 7.80 - 10.72 |

# n: amount of animals, %: seroprevalence, 95%CI: CI: Confidence interval

*In the departments of Ancash, the collection of bovine and goat serum samples could not be carried out due to logistical failures. In addition, in the departments of San Martín and Ucayali, goat samples could not be collected due to the absence.

**Supplementary 2 Table 2-4:**

The following three tables present the risk factors for bluetongue virus seroprevalence among cattle, sheep, and goats. The factors considered include age, sex, altitude, precipitation, relative humidity, and maximum temperature. These tables display the Odds Ratios (*OR*) calculated using simple and logistic regression. Separate analyses were conducted for each species of ruminant (Tables 2 - 4), and significance for all analyses was determined at a *p*-value of less than 0.05. These tables are shown as figures in the article.

| Variable | | Total n | Seroprevalence % | Simple regression | | | Multiple regression | | |
| --- | --- | --- | --- | --- | --- | --- | --- | --- | --- |
|  |  |  |  | *OR* | CI 95% | *p* | *OR* | CI 95% | *p* |
| Age | |  |  |  |  |  |  |  |  |
|  | > 6 months - ≤ 12 months | 264 | 17.80 | Ref. |  |  | Ref. |  |  |
|  | > 12 – ≤ 24 months | 682 | 17.01 | 0.95 | 0.61 – 1.47 | 0.804 | 1.02 | 0.54 – 1.91 | 0.957 |
|  | > 24 months | 2506 | 20.07 | 1.16 | 0.76 – 1.77 | 0.490 | 1.03 | 0.58 – 1.85 | 0.910 |
|  |  |  |  |  |  |  |  |  |  |
| Sex | |  |  |  |  |  |  |  |  |
|  | Female | 3013 | 20.1 | Ref. |  |  | Ref. |  |  |
|  | Male | 439 | 14.1 | 0.66 | 0.46 – 0.94 | 0.023 | 0.85 | 0.55 – 1.32 | 0.469 |
|  |  |  |  |  |  |  |  |  |  |
| Altitude (masl) | |  |  |  |  |  |  |  |  |
|  | ≤ 1,000 | 564 | 62.8 | Ref. |  |  | Ref. |  |  |
|  | > 1,000 – ≤ 2,000 | 398 | 46.5 | 0.52 | 0.29 – 0.92 | 0.025 | 1.30 | 0.67 – 2.54 | 0.432 |
|  | > 2,000 – ≤ 3,000 | 536 | 18.3 | 0.13 | 0.08 – 0.23 | < 0.001 | 0.77 | 0.32 – 1.88 | 0.569 |
|  | > 3,000 | 1954 | 1.5 | 0.01 | 0.00 – 0.02 | < 0.001 | 0.04 | 0.01 – 0.12 | < 0.001 |
|  |  |  |  |  |  |  |  |  |  |
| Precipitation (mm/day) | |  |  |  |  |  |  |  |  |
|  | 0 – ≤ 2 | 1595 | 15.1 | Ref. |  |  | Ref. |  |  |
|  | > 2 | 1857 | 22.9 | 1.68 | 1.09 – 2.59 | 0.019 | 2.90 | 1.70 – 4.97 | < 0.001 |
|  |  |  |  |  |  |  |  |  |  |
| Relative humidity (%) | |  |  |  |  |  |  |  |  |
|  | ≤ 60 | 758 | 3.6 | Ref. |  |  | Ref. |  |  |
|  | > 60 – ≤ 80 | 2220 | 17.5 | 5.73 | 2.78 – 11.85 | < 0.001 | 3.61 | 1.65 – 7.90 | 0.001 |
|  | > 80 | 474 | 53.0 | 30.47 | 13.50 – 68.80 | < 0.001 | 8.82 | 2.96 – 26.26 | < 0.001 |
|  |  |  |  |  |  |  |  |  |  |
| Maximum temperature (℃) | |  |  |  |  |  |  |  |  |
|  | ≤ 20 | 688 | 2.8 | Ref. |  |  | Ref. |  |  |
|  | > 20 – ≤ 30 | 2177 | 9.0 | 3.48 | 1.24 – 9.80 | 0.018 | 0.66 | 0.20 – 2.14 | 0.490 |
|  | > 30 | 587 | 76.8 | 116.74 | 40.79 – 334.16 | < 0.001 | 7.54 | 1.84 – 30.94 | 0.005 |

**Table 2.** Potential risk factors among cattle for BTV antibody detection in Peru, 2017-2019 by simple and multiple logistic regression

OR: Odds ratio, CI: Confidence interval, Ref: Reference group within the category, *p*: level of significance at 0.05

**Table 3.** Potential risk factors among sheep for BTV antibody detection in Peru, 2017-2019 by simple and multiple logistic regression

| Variable | | Total n | Seroprevalence % | Simple regression | | | Multiple regression | | |
| --- | --- | --- | --- | --- | --- | --- | --- | --- | --- |
|  |  |  |  | *OR* | CI 95% | *p* | *OR* | CI 95% | *p* |
| Age | |  |  |  |  |  |  |  |  |
|  | > 6 months - ≤ 12 months | 549 | 8.74 | Ref. |  |  | Ref. |  |  |
|  | > 12 – ≤ 24 months | 1039 | 7.60 | 0.86 | 0.53 - 1.39 | 0.537 | 0.87 | 0.58 - 1.32 | 0.527 |
|  | > 24 months | 1198 | 8.85 | 1.01 | 0.63 - 1.64 | 0.957 | 1.38 | 0.85 – 2.24 | 0.198 |
|  |  |  |  |  |  |  |  |  |  |
| Sex | |  |  |  |  |  |  |  |  |
|  | Female | 2262 | 8.7 | Ref. |  |  | Ref. |  |  |
|  | Male | 522 | 7.1 | 0.80 | 0.55 - 1.17 | 0.252 | 0.62 | 0.41 - 0.95 | 0.029 |
|  |  |  |  |  |  |  |  |  |  |
| Altitude (masl) | |  |  |  |  |  |  |  |  |
|  | ≤ 1,000 | 245 | 33.1 | Ref. |  |  | Ref. |  |  |
|  | > 1,000 – ≤ 2,000 | 133 | 15.0 | 0.36 | 0.14 - 0.92 | 0.033 | 0.66 | 0.19 - 2.28 | 0.510 |
|  | > 2,000 – ≤ 3,000 | 340 | 6.8 | 0.15 | 0.06 - 0.37 | < 0.001 | 0.67 | 0.18 - 2.56 | 0.562 |
|  | > 3,000 | 2068 | 5.3 | 0.11 | 0.06 - 0.21 | < 0.001 | 0.52 | 0.14 - 1.88 | 0.320 |
|  |  |  |  |  |  |  |  |  |  |
| Precipitation (mm/day) | |  |  |  |  |  |  |  |  |
|  | 0 – ≤ 2 | 2235 | 8.7 | Ref. |  |  | Ref. |  |  |
|  | > 2 | 551 | 6.9 | 0.77 | 0.35 - 1.74 | 0.534 | 0.97 | 0.49 - 1.91 | 0.928 |
|  |  |  |  |  |  |  |  |  |  |
| Relative humidity (%) | |  |  |  |  |  |  |  |  |
|  | ≤ 60 | 859 | 9.7 | Ref. |  |  | Ref. |  |  |
|  | > 60 – ≤ 80 | 1663 | 8.0 | 0.81 | 0.47 - 1.40 | 0.454 | 0.64 | 0.35 - 1.18 | 0.154 |
|  | > 80 | 264 | 6.4 | 0.64 | 0.20 - 2.07 | 0.458 | 0.89 | 0.32 - 2.29 | 0.761 |
|  |  |  |  |  |  |  |  |  |  |
| Maximum temperature (°C) | |  |  |  |  |  |  |  |  |
|  | ≤ 20 | 1486 | 4.9 | Ref. |  |  | Ref. |  |  |
|  | > 20 – ≤ 30 | 1125 | 6.8 | 1.4 | 0.79 - 2.50 | 0.252 | 1.26 | 0.53 - 2.98 | 0.598 |
|  | > 30 | 175 | 48.0 | 17.87 | 9.33 - 34.21 | < 0.001 | 12.57 | 3.31 - 47.73 | < 0.001 |

OR: Odds ratio, CI: Confidence interval, Ref: Reference group within the category, *p*: level of significance at 0.05

**Table 4.** Potential risk factors among goats for BTV antibody detection in Peru, 2017-2019 by simple and multiple logistic regression

| Variable | | Total n | Seroprevalence % | Simple regression | | | Multiple regression | | |
| --- | --- | --- | --- | --- | --- | --- | --- | --- | --- |
|  |  |  |  | *OR* | CI 95% | *p* | *OR* | CI 95% | *p* |
| Age | |  |  |  |  |  |  |  |  |
|  | > 6 months - ≤ 12 months | 347 | 12.39 | Ref. |  |  | Ref. |  |  |
|  | > 12 – ≤ 24 months | 699 | 9.73 | 0.76 | 0.42 – 1.37 | 0.361 | 0.48 | 0.26 - 0.87 | 0.016 |
|  | > 24 months | 522 | 6.32 | 0.48 | 0.19 – 1.19 | 0.113 | 0.44 | 0.17 - 1.10 | 0.079 |
|  |  |  |  |  |  |  |  |  |  |
| Sex | |  |  |  |  |  |  |  |  |
|  | Female | 1526 | 9.3 | Ref. |  |  |  |  |  |
|  | Male | 42 | 4.8 | 0.49 | 0.11 - 2.13 | 0.338 | 0.45 | 0.07 - 2.70 | 0.379 |
|  |  |  |  |  |  |  |  |  |  |
| Altitude (masl) | |  |  |  |  |  |  |  |  |
|  | ≤ 1,000 | 588 | 17.2 | Ref. |  |  | Ref. |  |  |
|  | > 1,000 – ≤ 2,000 | 230 | 15.7 | 0.89 | 0.51 - 1.58 | 0.699 | 0.83 | 0.42 - 1.61 | 0.574 |
|  | > 2,000 – ≤ 3,000 | 168 | 4.2 | 0.21 | 0.06 - 0.68 | 0.009 | 0.49 | 0.09 - 2.60 | 0.398 |
|  | > 3,000 | 582 | 0 | - | - | - | - | - | - |
|  |  |  |  |  |  |  |  |  |  |
| Precipitation (mm/day) | |  |  |  |  |  |  |  |  |
|  | 0 – ≤ 2 | 1538 | 9.1 | Ref. |  |  | Ref. |  |  |
|  | > 2 | 30 | 13.3 | 1.54 | 0.30 - 7.87 | 0.603 | 1.45 | 0.28 - 7.41 | 0.654 |
|  |  |  |  |  |  |  |  |  |  |
| Relative humidity (%) | |  |  |  |  |  |  |  |  |
|  | ≤ 60 | 468 | 12.8 | Ref. |  |  |  |  |  |
|  | > 60 – ≤ 80 | 990 | 8.1 | 0.60 | 0.31 - 1.16 | 0.130 | 0.63 | 0.32 - 1.25 | 0.182 |
|  | > 80 | 110 | 3.6 | 0.26 | 0.05 - 1.28 | 0.097 | 0.75 | 0.08 - 7.45 | 0.808 |
|  |  |  |  |  |  |  |  |  |  |
| Maximum temperature (°C) | |  |  |  |  |  |  |  |  |
|  | ≤ 20 | 462 | 0.4 | Ref. |  |  | Ref. |  |  |
|  | > 20 – ≤ 30 | 652 | 4.5 | 10.77 | 2.57 - 45.12 | 0.001 | 0.24 | 0.02 - 3.78 | 0.309 |
|  | > 30 | 454 | 24.9 | 76.66 | 15.94 - 368.74 | < 0.001 | 1.13 | 0.04 - 29.96 | 0.942 |

OR: Odds ratio, CI: Confidence interval, Ref: Reference group within the category, *p*: level of significance at 0.05
